# FAM111B knockdown attenuates tumorigenesis of ovarian cancer via the downregulation of MYC

**DOI:** 10.1186/s12885-025-14740-6

**Published:** 2025-08-09

**Authors:** Guoyu Yu, Fang Wei, Wanying Li, Qiuyun Guo, Lihong Zhang

**Affiliations:** 1 Department of Oncology, Tongji Hospital, Tongji Medical College, Huazhong University of Science and Technology, Wuhan, China; 2 Department of Oncology, Xiangyang No.1 People’s Hospital, Hubei University of Medicine, Xiangyang, China

**Keywords:** FAM111B, Ovarian cancer, Tumorigenesis, MYC, Prognosis

## Abstract

**Background:**

Ovarian cancer, a prevalent malignancy with the highest fatality rate among gynecological cancers, continues to face challenges in the development of effectively targeted therapeutic approaches. While the FAM111B gene has been implicated in various cancer types, its specific role in ovarian cancer remains poorly understood.

**Methods:**

The ES2 and A2780 ovarian cell lines were exploited to explore the cellular proliferation, migration, invasion, and epithelial-mesenchymal transition (EMT) in FAM111B knockdown experiments. We constructed a mouse tumor model to investigate the impact of FAM111B silencing in vivo; employed tissue microarray to explore the prognostic value of different FAM111B expression levels; and applied western-blot assay, MYC-overexpression rescue experiments, protein transcriptomics, and bioinformatics analysis to examine the downstream molecular mechanisms underlying FAM111B actions.

**Results:**

Our in vitro experiments indicated that the inhibition of FAM111B resulted in reduced cellular proliferation, migration, invasion, and EMT in ovarian cancer cell lines and in the suppression of tumor growth in a mouse xenograft model. Immunohistochemical analyses conducted on tissue-microarray samples obtained from patients with serous ovarian cancer indicated that elevated levels of FAM111B expression were associated with unfavorable prognostic outcomes. The silencing of FAM111B mechanistically constrained MYC expression, with subsequent MYC overexpression reversing the phenotypic suppression caused by FAM111B silencing. Additionally, protein transcriptomic analysis implicated FAM111B in genetic-information processing via the MYC pathway, underscoring FAM111B’s central role in ovarian cancer tumorigenesis.

**Conclusions:**

These findings suggest that FAM111B may serve as a novel biomarker and potential therapeutic target in ovarian cancer.

**Supplementary Information:**

The online version contains supplementary material available at 10.1186/s12885-025-14740-6.

## Introduction

Ovarian cancer is one of the most prevalent cancers, and it possesses the fifth highest fatality rate among women [1]. There are currently only a limited number of targeted therapies—including poly ADP-ribose polymerase (PARP) inhibitors and anti-vascular monoclonal antibodies—that are established as standard treatments for ovarian cancer; however, their effectiveness is equivocal [2]. Hence, there is an urgent requirement for the identification of novel therapeutic targets in the treatment of ovarian cancer.

The human family with sequence similarity 111 member B (FAM111B) gene, located on chromosome 11q12.1, encodes a protein with a C-terminal trypsin-like cysteine/serine peptidase domain. Initially FAM111B is identified as the causative gene for hereditary fibrosing poikiloderma (POIKTMP), this condition is characterized by tendon contractures, myopathy, and pulmonary fibrosis [3, 4]. In 2020, researchers elucidated the physiological mechanism of FAM111B at the molecular level. Through interactive analysis, these investigators identified FAM111B as a potential binding partner for FAM111A. Mutations in FAM111B were found to enhance its proteolytic enzyme activity, thereby enhancing the protease activity of FAM111A. This cascade of events ultimately inhibits DNA and RNA synthesis, resulting in heightened apoptosis and disruption of microtubule network integrity and cellular adaptability [5].

FAM111B has been identified as an oncogene in several types of cancer, including pancreatic adenocarcinoma, lung adenocarcinoma, bladder cancer, and hepatocellular carcinoma [6–10]. 12% of patients with the POIKTMP syndrome are documented to have pancreatic cancer without any discernible additional risk factors (2 out of 17; 12%) [6]. The upregulation of the FAM111B protein is identified in specific subtypes of lung adenocarcinoma such as papillary-predominant adenocarcinoma and is correlated with unfavorable overall survival outcomes [11, 12]. Nevertheless, the precise role of FAM111B remains unclear, and its potential involvement in the pathogenesis of ovarian cancer has yet to be established conclusively.

In this study, we examined the role of FAM111B in ovarian cancer through in vitro and in vivo experiments, and our analysis of FAM111B protein levels in tissue microarray samples revealed a correlation between elevated FAM111B expression and unfavorable patient outcomes. Mechanistic investigations indicated that FAM111B facilitated malignant progression of cancer cells by modulating MYC. These findings suggest that FAM111B exhibits potential as a prognostic marker and therapeutic target for enhancing the efficacy of ovarian cancer treatment.

## Materials and methods

### Cell culture

ES-2 and A2780, two commonly used ovarian cancer cell lines, were procured from the American Type Culture Collection (ATCC) located in Manassas, VA, USA. ES-2 cells were maintained in DMEM medium (Corning, USA), while A2780 cells were cultured in RPMI 1640 medium (Corning, USA), both supplemented with 10% fetal bovine serum (Ausbian, Australia). The cells were incubated under standard conditions in a 5% CO_2_ atmosphere at 37 °C.

### Lentiviral transfection

The shRNA targeting sequences deployed in this study were derived from the NCBI reference accession NM_198947, with the sense sequence 5’-GCCTGCCTAGTGATTCTCATT-3’ and the anti-sense sequence 5’-AATGAGAATCACTAGGCAGGC-3’. Additionally, the inserted shRNA nonsense sequence employed was 5’-TTCTCCGAACGTGTCACGT-3’. Upon reaching a plating volume of approximately 30%, the cells were transfected for a duration of 12 h with either FAM111B-targeting shRNA (shFAM111B) or a nonsense control shRNA (shCtrl) lentivirus, and co-transfected in combination with MYC-overexpressing vectors (LV-MYC(44590-1)) or mock control vectors (LV-CON294), adopting an optimal multiplicity of transfection of 10 TU/cell. Approximately 72 h post-infection, the fluorescence rate of green fluorescent protein exceeded 70–80%, and cell confluency reached approximately 80%, as observed under a fluorescence microscope (Olympus, Japan). We subsequently harvested the cells for further downstream experiments.

### Cell-proliferation assay

At 72 h post-transfection, a total of 2000 cells were plated in each well of 96-well plates. Subsequently, 20 µL of MTT reagent at a concentration of 5 mg/mL was introduced, followed by a 4-hour incubation period at 37 °C. The culture medium was then aspirated, and 100 µL of DMSO was added to each well. We then measured the optical density (OD) at 490/570 nm to assess cell viability. To quantify cellular proliferation, green-fluorescent cells were directly counted using the Celigo Image Cytometer (Nexcelom, USA).

### Colony-formation assay

Transfected cells were plated in six-well plates at a density of 1000 cells per well and maintained in culture with medium refreshed every 3 days. After 9 days, colonies were fixed in 4% paraformaldehyde for 30 min, followed by staining with 0.1% crystal violet for 15 min. The colonies were subsequently quantified and imaged.

### Cell-migration and -invasion assay

In the migration assay, 150 µL of cellular suspension containing approximately 4 × 10^5^ cells in serum-free medium was introduced into the upper chamber (Corning, USA). Subsequently, 600 µL of medium containing 30% FBS was introduced into the lower chamber, and the plates were then incubated at 37 °C for a duration of 20 h. Following this incubation period, the cells were fixed on the filter surface using alcohol for a period of 30 min, after which crystal violet was applied for 15 min. Five random fields per filter were then selected for image capture at a magnification of 200X, and the cells that had migrated or invaded into the lower chamber were quantified and subjected to statistical analysis.

The invasion assay was conducted by preparing an upper chamber containing a Matrigel-coated membrane. This involved rehydrating the Matrigel matrix layer by adding 500 µL of serum-free medium to both the upper and lower chambers, followed by an incubation at 37 °C for 2 h. The medium was then replaced with 200 µL of cell suspension (approximately 4 × 10^5^ cells in serum-free medium) in the upper chamber and 750 µL of medium containing 30% FBS in the lower chamber. The plates were ultimately incubated at 37 °C for 60 h. Fixing and staining procedures were conducted as previously described.

### Western-blot analysis

Cells were gathered and lysed in RIPA Lysis Buffer (Thermo, USA) supplemented with 1% PMSF and 1 mmol/L NaF at a temperature of 4 °C, and protein concentrations were determined using a BCA Protein Assay Kit (Servicebio, China). 5x-Loading buffer was introduced, and the samples were subjected to boiling for 10 min. The samples underwent electrophoresis on 10% SDS-PAGE separation gels and were subsequently transferred to PVDF membranes at 4 °C, applying a constant current of 300 mA for a duration of 150 min. Following a 2-hour blocking period with 5% non-fat milk at 25 °C, the primary antibody (FAM111B, NBP1-86645,Novus bio, USA) was applied and left to incubate overnight at 4 °C, followed by a 1-hour incubation with the secondary antibody (see Supplementary Table 1). To detect multiple proteins in one batch of samples, some membranes were cut according to their respective molecular weights prior to undergoing hybridization with antibodies for the purpose of detecting alterations in multiple proteins on a single membrane. We employed the ECL method with the West Pico Chemiluminescent Substrate (Thermo, USA) for color development, and gray values were quantified using ImageJ software (NIH, USA).

### Xenograft study

Four-week-old female BALB/c nude mice were purchased from Shanghai Lingchang Biological Technology Co. Ltd (Shanghai, China). The mice were housed and managed in compliance with the regulations set forth by the Animal Care and Use Committee within a conventional laboratory environment. A total of 18 mice were selected and randomly divided into shFAM111B group or shCtrl group. ES-2 cells were transfected with either shRNA-FAM111B or a shRNA-negative control lentiviral vector, as described in the previous lentiviral transfection section. After a 72-hour post-transfection incubation period, 1.0 × 10^7^ actively proliferating cells were subcutaneously implanted into the right axilla of the experimental subjects. Tumor volume was measured every 2–3 days using the formula (length × width^2^) × 0.52, beginning 2 weeks after the initial injections. The mice were euthanized 26 days post-injection using an intraperitoneal injection of 3% pentobarbital sodium at a dosage of 150 mg/kg body weight. We subsequently cervically dislocated the mice to verify cessation of their vital signs. Finally, xenograft tumors were collected, measured, and weighed.

### Tissue microarrays and patient cohort

The tissue microarrays purchased from Shanghai Outdo Biotech Company (Array No. HOvarianaC151Su01) consisted of 78 tumor tissues from serous ovarian cancer patients. These samples were obtained from patients, who were pathologically diagnosed with serous ovarian cancer after primary surgery between 2009 and 2013. The mean age was 53 years (range 23–75 years). According to the UICC/AJCC TNM clinical stage classification system, 20 cases were stage I-II, and 58 cases were stage III-IV. During the median follow-up time of 51 months, the survival time was range from 4 to 109 months.

### Immunohistochemical (IHC) analysis

IHC analysis was performed on formalin-fixed paraffin-embedded tissue slices. The expression of FAM111B was detected using anti-FAM111B antibody at a dilution of 1:300 with an automatic immunohistochemical staining instrument (Dako, Autostainer Link48, Denmark). Following the process of drying the slices, dewaxing, and retrieving antigens, the primary and secondary antibody solutions were introduced for antibody incubation. The samples underwent color coating, development, re-staining with hematoxylin, and sealing. Each section was assessed by two pathologists individually and scored utilizing the Aperio Image Scope version 11 system. We evaluated FAM111B expression levels based on the positivity rate and staining intensity (0, negative; 1, weak; 2, moderate; 3, strong). The staining intensity and positivity rate were evaluated independently in both the cytoplasm and nucleus [13, 14]. The IHC score for the nucleus was calculated as the product of the nuclear intensity score and the percentage of cells staining positively. The IHC score for the cytoplasm was likewise determined by multiplying the cytoplasmic intensity score by the percentage of positively staining cells [15]. We ultimately calculated the overall IHC score as the combined total of the individual nuclear and cytoplasmic scores.

### Proteomic analysis

ES-2 cells were transfected with either FAM111B-targeting shRNA (shFAM111B, shFAM) or control shRNA (shCtrl) lentivirus and then harvested after a 72-hour incubation period. We conducted the label-free quantitative proteomic analysis by adding SDT buffer (4% SDS, 100 mM Tris-HCl, pH 7.6) to the cells individually, followed by protein quantification using the BCA Protein Assay Kit (Beyotime, China). Subsequently, 20 µg of protein from each sample was separated on a 12% SDS-PAGE gel and stained with Coomassie Blue R-250. The protein solution underwent filter-aided sample preparation (FASP) for proteolytic enzymolysis, and the proteins were analyzed using Orbitrap Exploris 480 mass spectrometry (Thermo, USA). Maxquant software was employed for the identification of peptide features in the raw data, while protein data were obtained via the data-dependent acquisition (DDA) method. We analyzed the mass- spectrometric data for protein characterization using the protein sequence database at http://www.uniprot.org.

### Bioinformatics analysis

The mRNA sequences were obtained from 376 serous ovarian cancer tissues and 180 normal ovarian tissues by accessing data from The Cancer Genome Atlas (TCGA) and Genotype-Tissue Expression (GTEx) databases. The TCGA database can be found at https://portal.gdc.cancer.gov, and the GTEx database can be found at https://www.gtexportal.org/home/. The mRNA stemness indices (mRNAsi) were initially computed by exploiting the OCLR algorithm to investigate the correlation between FAM111B and the malignancy index (tumor stemness score, mRNAsi) of the cancer cells. We employed the ssGSEA algorithm via the R software “GSVA” package to examine the potential pathways associated with FAM111B. Finally, the “pRRophetic” R package was implemented to determine the IC50 in individual ovarian cancer patients by accessing the pharmacogenomics database of the Genomics of Drug Sensitivity in Cancer (GDSC: https://www.cancerrxgene.org/). Furthermore, functional enrichments for genes related to FAM111B were conducted through Gene Ontology (GO) analysis and Kyoto Encyclopedia of Genes and Genomes (KEGG) analysis, using an online platform (https://www.bioinformatics.com.cn) for data analysis and visualization.

### Statistics analysis

Group differences were evaluated using a two-sided *t* test, ANOVA, or Chi-squared test, with significance defined at *P* < 0.05. We conducted survival analysis using Kaplan-Meier statistics. Statistical analysis was performed using R software v4.0.3 (R Foundation for Statistical Computing, Vienna, Austria) and GraphPad Prism (version 7.0), and P-values were calculated to assess statistical significance and are represented in the figures as follows: *, *P* < 0.05; **, *P* < 0.01; ***, *P* < 0.001; ****, *P* < 0.0001.

## Results

### FAM111B silencing inhibits proliferation of ovarian cancer cells

**Fig. 1:**
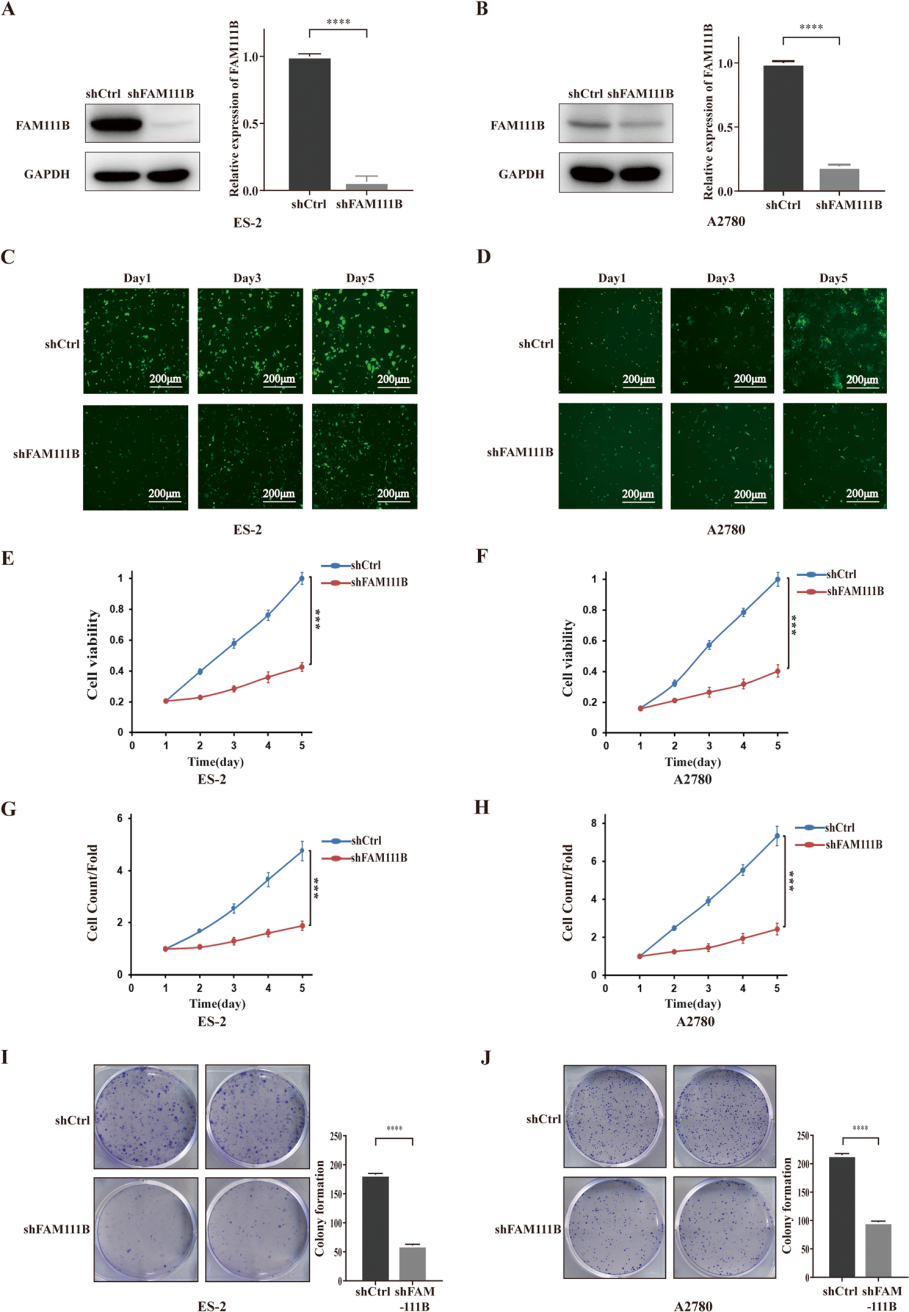
FAM111B silencing inhibits proliferation of ovarian cancer cells. (A–B) FAM111B protein levels were detected by western blots in the shControl (shCtrl) and shFAM111B groups in ES2 and A2780 cell lines. (C–D) Fluorescence images of cellular proliferation labeled by green fluorescent protein on days 1, 3, and 5. (E–F) Cellular viability was evaluated with MTT assay. (G–H) The number of proliferative cells in the ES-2 and A2780 cell lines was quantified through direct counting using the Celigo Image Cytometer. (I–J) Clonogenicity of shCtrl and shFAM111B groups in ES-2 and A2780 cells

To investigate the impact of FAM111B on the proliferation of ovarian cancer, shRNA lentiviral vectors were deployed to induce FAM111B knockdown in ES-2 and A2780 ovarian cancer cells. The results depicted in Fig. 1A–D show a significant diminution in FAM111B expression. Subsequent MTT assay analysis revealed a decrease in cellular viability in both ES-2 and A2780 cells following FAM111B silencing (shFAM111B) (Fig. 1E–F). Furthermore, utilizing the Celigo Image Cytometer platform, we noted that the number of cells was markedly reduced upon FAM111B silencing (Fig. 1G–H). Furthermore, the suppression of FAM111B resulted in a decrease in the colony-formation capacity of the cells, as illustrated in Fig. 1I–J. Taken together, these findings indicate that the inhibition of FAM111B hinders the viability and proliferation of ovarian cancer cells.

### FAM111B silencing suppresses the migration and invasion of ovarian cancer cells

**Fig. 2:**
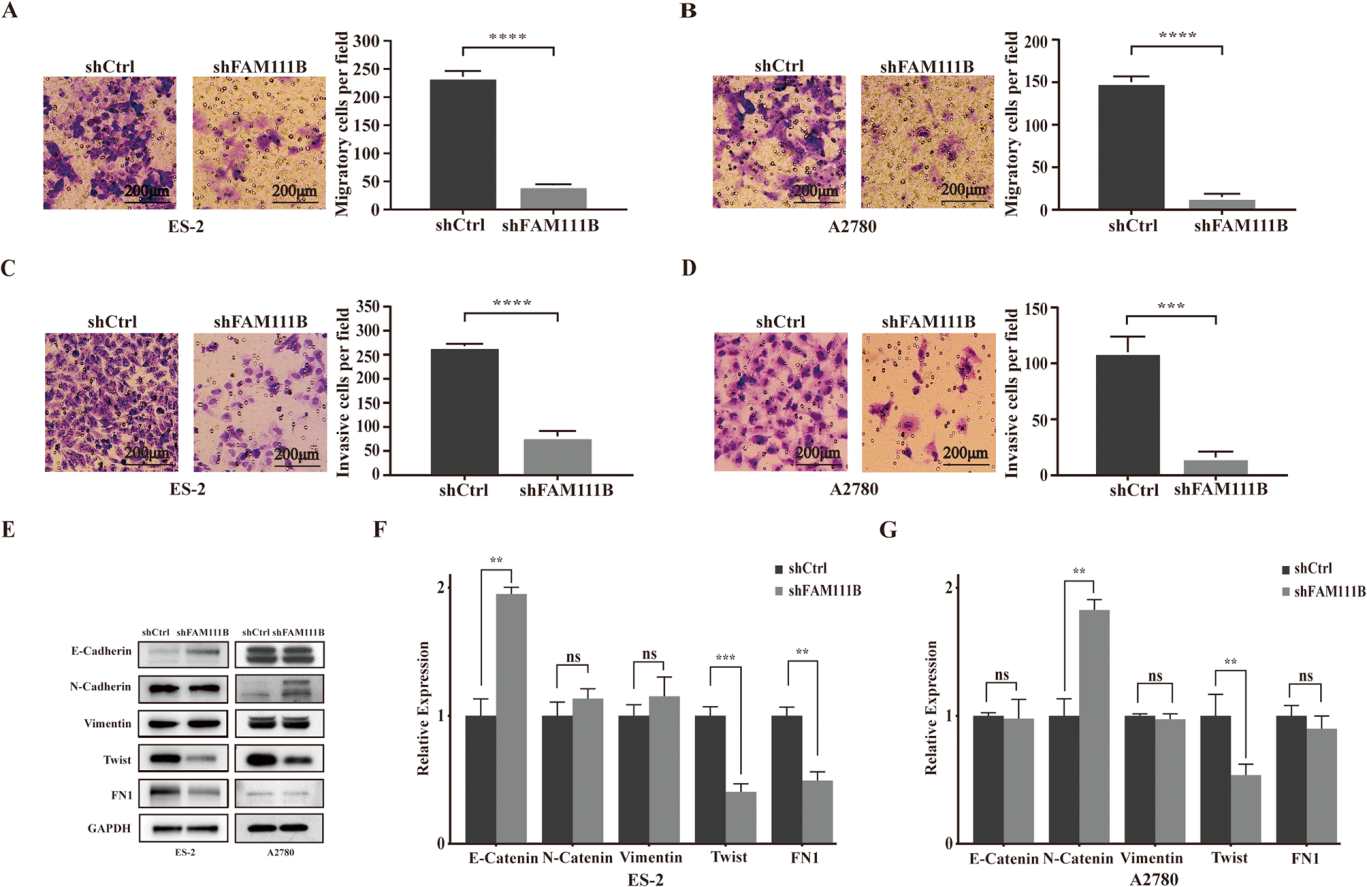
FAM111B silencing suppresses the migration, invasion, and epithelial- mesenchymal transition. (A–B) In the migration assay, the number of migratory cells in the shFAM111B group was significantly reduced compared to the shCtrl group. (C–D) In the invasion assay, the number of invasive cells in the shFAM111B group was significantly reduced compared to the shCtrl group. (E) Western blot analysis of EMT-related factors in both the shCtrl and shFAM111B groups in ES-2 and A2780 cells. (F–G) Quantitative analysis of EMT-related protein expression was performed based on the western blot bands

To assess the impact of FAM111B on the migratory and invasive properties of ovarian cancer cells, we conducted Transwell assays; and our results indicated a notable decrease in the migratory capacity of FAM111B-silenced cells compared to the shCtrl group (Fig. 2A and B). Additionally, the invasive potential of the cells was hindered upon FAM111B silencing, as evidenced by reduced penetration of an intercompartmental semipermeable Matrigel membrane (Fig. 2C and D). Collectively, these findings suggest that the downregulation of FAM111B exerts a suppressive action on the migratory and invasive capabilities of ES-2 and A2780 cells.

### FAM111B silencing may inhibit the EMT

To examine the potential role of FAM111B in facilitating the EMT process during tumor progression, we assessed the expression levels of key EMT biomarkers in ES-2 and A2780 cells. Our findings, illustrated in Fig. 2E–G, indicate that knockdown of FAM111B in ES-2 cells led to an increase in E-cadherin, a known suppressor of EMT, while no significant changes were observed in the levels of the mesenchymal markers N-cadherin or vimentin. These findings suggest that the silencing of FAM111B hinders tumor cellular adhesion, invasion, and EMT progression. Conversely, in A2780 cells, we observed an upregulation of the mesenchymal marker N-cadherin. Additionally, the expression of key transcription factors involved in the EMT regulatory network (such as Twist and FN1) was suppressed in the shFAM111B group compared to the shCtrl group. These results support the hypothesis that FAM111B silencing impedes EMT progression by downregulating EMT-related transcription factors, including Twist and FN1.

### MYC overexpression reverses the phenotypic suppression mediated by FAM111B silencing

**Fig. 3:**
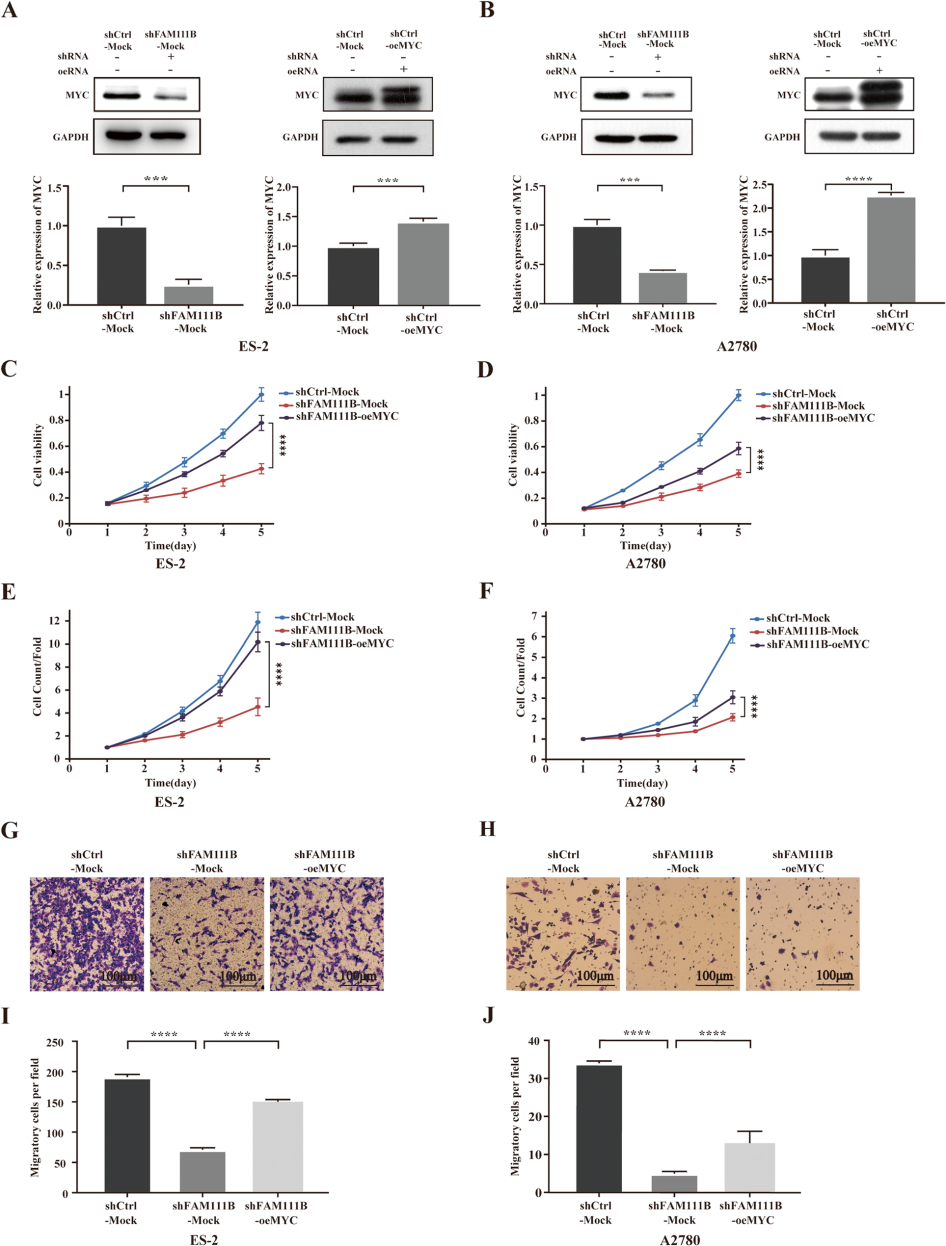
MYC overexpression reverses the phenotypic suppression mediated by FAM111B silencing. (A–B) Western blot analysis of MYC expression under various conditions, including FAM111B knockdown (shFAM111B) versus control (shCtrl), and MYC overexpression (oeMYC) versus mock (Mock). (C–D) Assessment of cellular viability across shCtrl-Mock, shFAM111B-Mock, and shFAM111B-oeMYC groups. (E–F) Quantification of proliferative cell numbers in shCtrl-Mock, shFAM111B-Mock, and shFAM111B-oeMYC cells. (G–H) Representative images from migration assays conducted on shCtrl-Mock, shFAM111B-Mock, and shFAM111B-oeMYC cells. (I–J) Enumeration of migrating cells within the shCtrl-Mock, shFAM111B-Mock, and shFAM111B-oeMYC groups

MYC is one of the most extensively analyzed nuclear oncogenes and is critical to the regulation of cell growth, proliferation, and tumorigenesis [16]. Thus, we herein posited the potential involvement of MYC in the regulation of the phenotype of FAM111B. To investigate this, we used western blotting on MYC in cells with either shCtrl or shFAM111B and found a notable decrease in MYC expression in cells with FAM111B knockdown (Fig.3A and B). And we performed co-transfection experiments involving a control (Mock) or MYC-overexpressing lentiviral vector (oeMYC) in shFAM111B and shCtrl cells. ShFAM111B-oeMYC cells exhibited an elevated rate of cell proliferation compared to shFAM111B-Mock cells (Fig. 3C–F), indicating a potential reversal of the inhibitory effects on proliferation as mediated by FAM111B silencing through MYC overexpression. The migratory capacity was consistently found to be reinstated in shFAM111B-oeMYC cells relative to shFAM111B-Mock cells (Fig. 3G–J). Our findings suggest that the overexpression of MYC counteracts the phenotypic inhibition induced by FAM111B knockdown. Thus, FAM111B appears to facilitate proliferation, invasion, and migration by modulating MYC.

### FAM111B silencing inhibits in vivo tumor growth

**Fig. 4:**
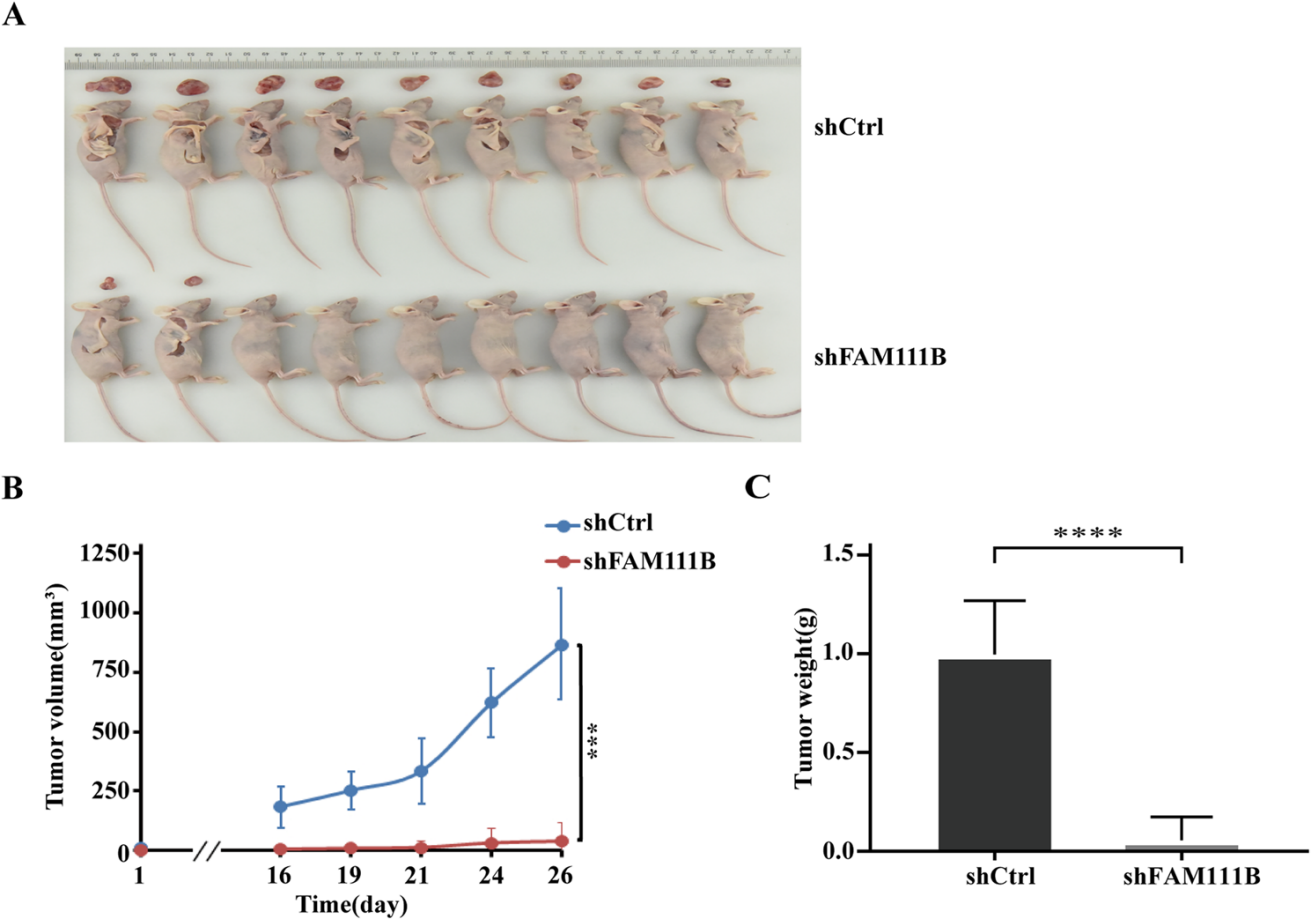
FAM111B silencing inhibits in vivo tumor growth. (A) Images of xenograft tumors 26 days after injection; (B) Tumor growth as determined using calipers; (C) Tumor weight 26 days post-injection. Experiments were performed in BALB/c nude mice harboring shCtrl or shFAM111B ES-2 cells

To assess the impact of FAM111B silencing on tumor growth in vivo, we established a BALB/c nude mouse xenograft model with shFAM111B or shCtrl ES-2 cells. The xenograft tumors retrieved from the mice manifested smaller or non-existent growth in the shFAM111B group compared to the shCtrl group (Fig. 4A). The decelerated growth pattern of the shFAM111B group was further validated through regular measurements of tumor volume every 2 or 3 days commencing on day 26 post-injection, as illustrated in Fig. 4B; additionally, the final weights of the shFAM111B group were notably decreased (Fig. 4C). These findings indicate that silencing of FAM111B exerts an inhibitory impact on tumor growth in vivo, congruent with our observations of FAM111B’s role in promoting the malignant phenotype in vitro.

### FAM111B is highly expressed and indicates a poor prognosis in serous ovarian cancer

**Fig. 5:**
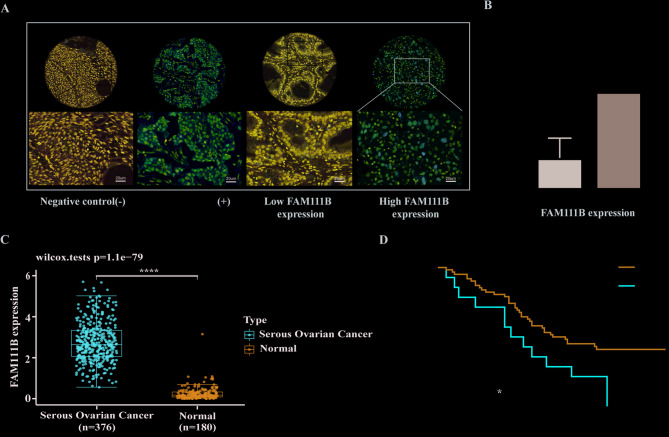
Elevated expression of FAM111B is related to poor patient prognosis. (**A**) Immunohistochemical (IHC) staining of serous ovarian cancer tissues. Panels (i) and (ii) represent the negative and positive controls for FAM111B expression, respectively. Panels (iii) and (iv) display representative samples with low and high expression of FAM111B, respectively. (**B**) IHC scores between the FAM111B low- and high-expression groups demonstrated statistical significance (*, *P* < 0.05; **, *P* < 0.01; ***, *P* < 0.001; ****, *P* < 0.001). (**C**) Statistical analysis of FAM111B expression in the TCGA and GTEx databases. (**D**) Overall survival (OS) curves for serous ovarian cancer patients stratified by low and high FAM111B expression levels

To investigate the expression status and prognostic significance of FAM111B in ovarian cancer, we utilized tissue microarrays for IHC detection, categorizing FAM111B into high- and low-expression groups (Fig.5A); and analysis of the IHC scores revealed a significantly elevated expression of FAM111B in the high-expression group compared to the low-expression group (Fig. 5B). Combining the data on serous ovarian cancer and normal tissue samples from TCGA and GTEx databases revealed a significantly elevated level of FAM111B mRNA in serous ovarian cancer tissues compared to that in normal tissues (Fig. 5C). Survival analysis of 78 cases of serous ovarian cancer from tissue microarrays indicated that patients with high FAM111B expression possessed a significantly shorter median overall survival time compared to those with low expression (38 vs. 58 months; HR, 2.529; 95% CI, 1.110–5.762) (Fig. 5D). These findings indicated that FAM111B may serve as a prognostic indicator of poor clinical outcomes in patients with serous ovarian cancer.

### FAM111B is involved in genetic-information processing through the MYC pathway

**Fig. 6:**
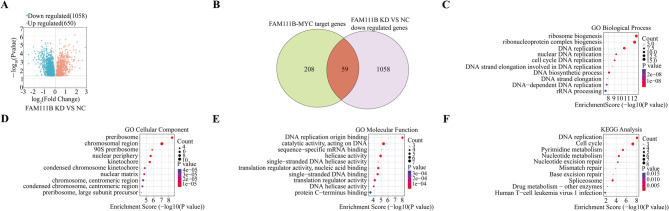
Functional enrichment analysis of FAM111B–MYC-related genes. Volcano plot illustrating the upregulation and downregulation of genes in FAM111B knockdown (KD) and control (NC) groups; (B) Venn diagram depicting the overlap between FAM111B- and MYC-targeted genes; (C) Gene Ontology (GO) analysis of biological processes; (D) GO analysis of cellular components; (E) GO analysis of molecular functions; (F) Kyoto Encyclopedia of Genes and Genomes (KEGG) pathway analysis

**Fig. 7:**
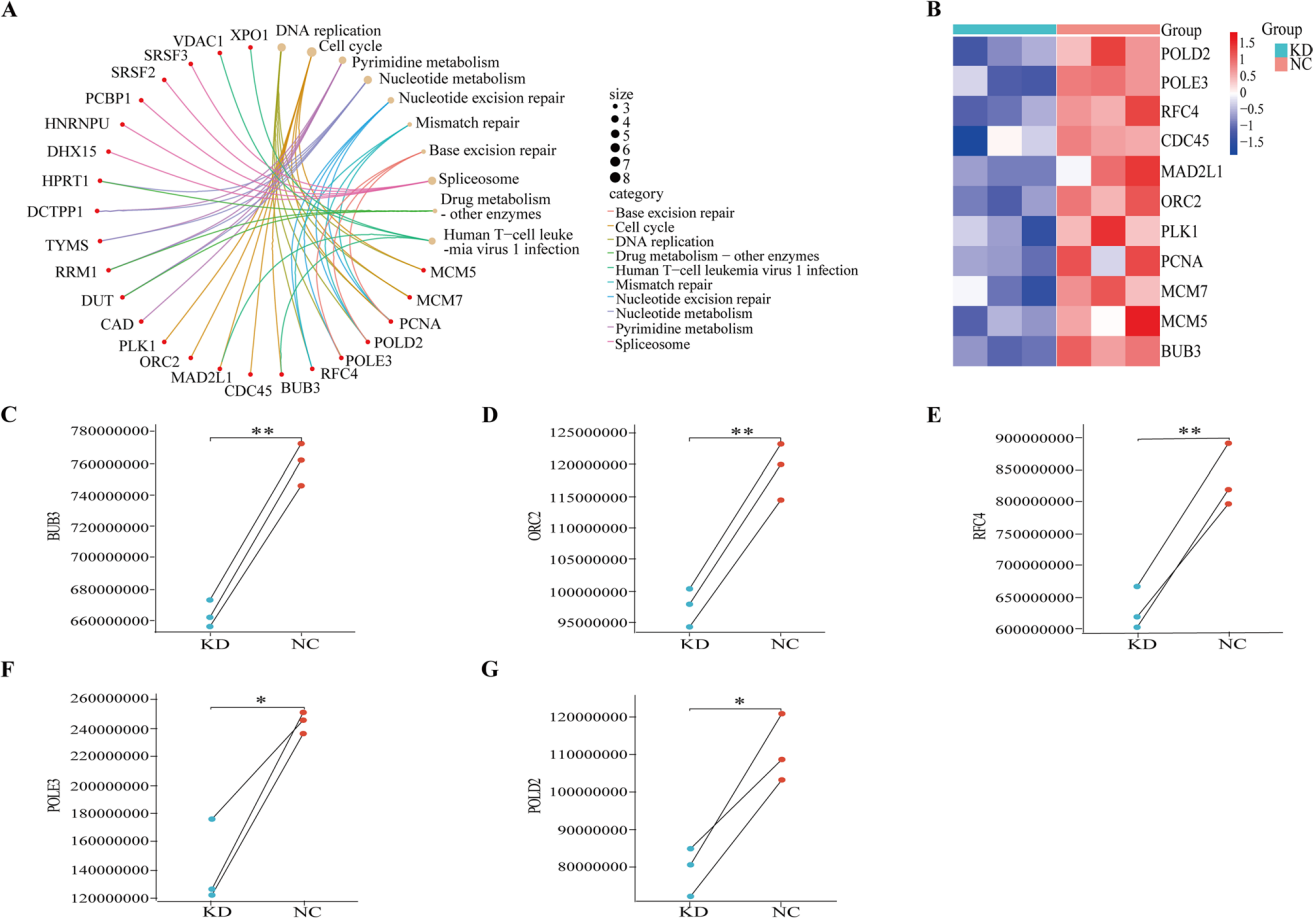
Genes related to genetic-information processing. (A) Genes in KEGG analysis; (B) Heatmap of genetic information processing-related genes; (C–G) Differential-expression pair plot of BUB3, ORC2, RFC4, POLE3, and POLD2 genes in FAM111B knockdown (KD) and control (NC) groups

The protein transcriptomic data associated with FAM111B were analyzed using a volcano plot (Fig. 6A), and our analysis revealed 650 upregulated proteins and 1058 downregulated proteins in the FAM111B knockdown (KD) and control groups, as determined by fold-change greater or less than 1 and a significance level of *P* < 0.05. We identified 208 genes that were enriched and associated with both the MYC pathway and the FAM111B gene, and these 208 FAM111B-MYC-related genes were predicted using bioinformatic technology based on data from the TCGA database. Subsequently, 1058 downregulated genes were identified in both the FAM111B KD and control groups through protein-transcriptomic experiments. By taking the intersection of these gene sets, we derived a final set of 59 genes that were under the regulation of the FAM111B-MYC axis (Fig. 6B). The GO and KEGG enrichment analysis of 59 genes identified a significant enrichment of FAM111B-MYC-related genes in genetic-information processing, as illustrated in Fig. 6C–F. The results of the KEGG analysis indicated a close association between FAM111B-MYC-related genes and various processes related to genetic-information processing, including DNA replication, cell-cycle regulation, nucleotide excision repair, mismatch repair, and base excision repair pathways. Eleven genes enriched in this pathway—RFC4, POLE3, POLD2, PLK1, PCNA, ORC2, MCM7, MCM5, MAD2L1, CDC45, and BUB3 (Fig. 7A)—were identified, and their expression patterns are visually presented in heatmaps (Fig. 7B). Significant differences in the expression levels of BUB3, ORC2, RFC4, POLE3, and POLD2 were also verified between the FAM111B KD and control groups in our proteomics data (Fig. 7C–G).

### Bioinformatics analysis reconfirm the function of FAM111B in the MYC pathway

**Fig. 8:**
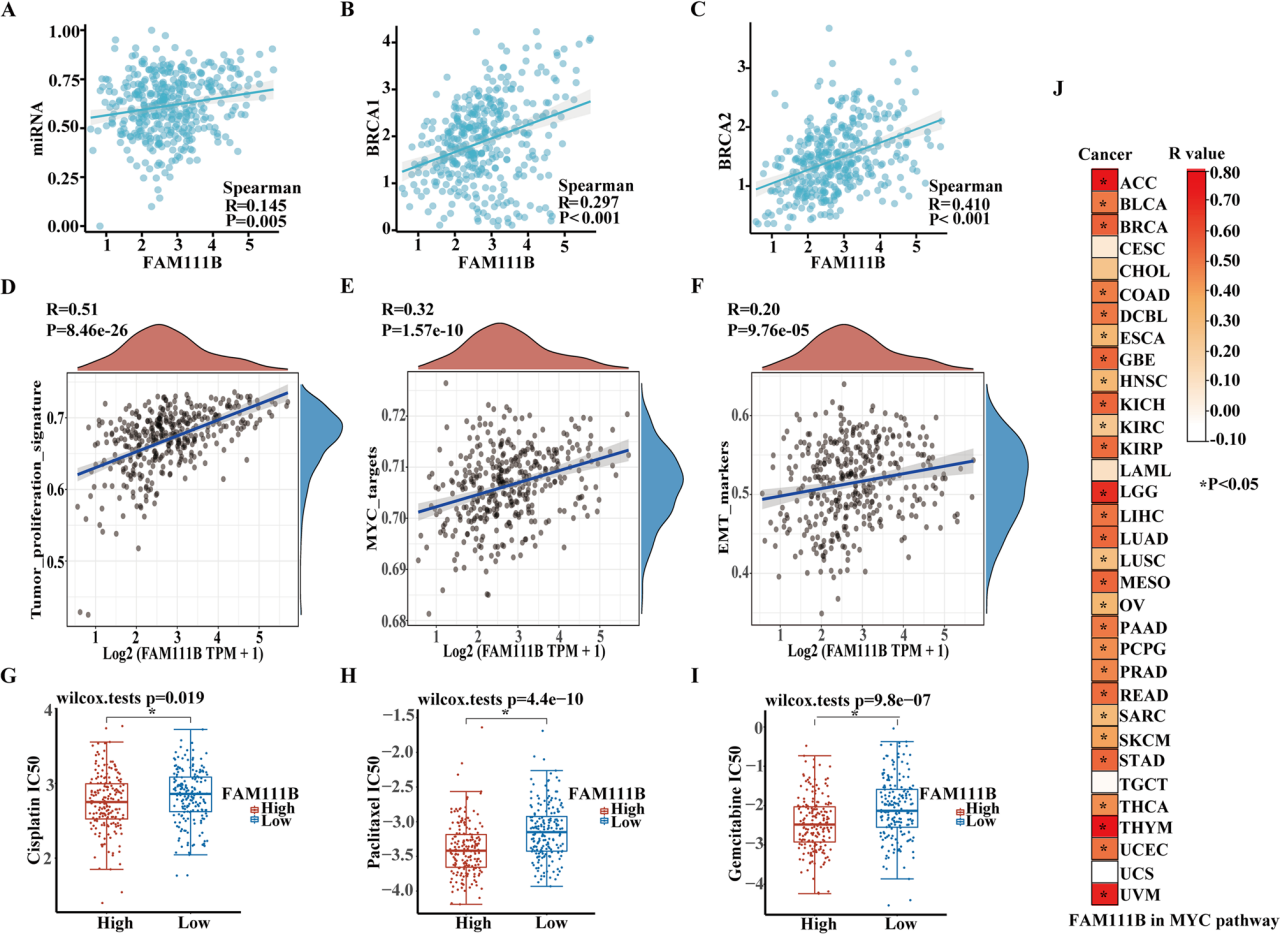
Bioinformatics analysis of FAM111B in the TCGA database

Utilizing data from the TCGA database, our analysis revealed a positive correlation between FAM111B and the cellular malignancy index mRNAsi (*R* = 0.145, *P* = 0.005) (Fig. 8A); in addition, FAM111B exhibited positive correlations with the key molecules BRCA1 (*R* = 0.297, *P* < 0.001) and BRCA2 (*R* = 0.410, *P* < 0.001) (Fig. 8B–C). Further investigation into pathways demonstrated that FAM111B was linked to the tumor proliferation signature (*R* = 0.51, *P* < 0.001), MYC pathway (*R* = 0.32, *P* < 0.001), and the EMT (*R* = 0.20, *P* < 0.001) in ovarian cancer (Fig. 8D–F). Based on IC50 values, we hypothesized that cisplatin, paclitaxel, and gemcitabine enhanced therapeutic efficacy in patients exhibiting a high-expression phenotype of FAM111B (Fig. 8G–I). An analysis of 33 different tumor types within the TCGA database also indicated a significant association between FAM111B and the MYC pathway in the majority of tumors (28/33) (*P* < 0.05) (Fig. 8J). This observation strongly suggests that FAM111B plays a crucial role in various tumor types in the promotion of malignant proliferation and metastasis of cancer cells via the MYC-signaling pathway.

(A–C) Correlation between FAM111B and mRNA stemness indices for BRCA1 and BRCA2; (D–F) Signaling pathways involved in FAM111B based on ssGSEA; (G–I) Prediction of therapeutic drug efficacy for FAM111B; (J) Correlation between FAM111B and the MYC pathway based on analysis of all 33 types of tumors.

## Discussion

In summary, our findings reveal that FAM111B functions in ovarian carcinogenesis via the MYC pathways. Specifically, downregulation of FAM111B led to a notable decrease in ovarian cell growth and proliferation both in vitro and in vivo. Additionally, the elevated FAM111B expression was associated with poor prognosis of serous ovarian cancer patients. Furthermore, the depletion of FAM111B led to the downregulation of MYC, while in rescue experiments, the overexpression of MYC could reverse the phenotype caused by FAM111B knockdown. Subsequent protein transcriptomic analysis into the mechanism of action revealed that the FAM111B-MYC axis was important in promoting malignant proliferation by regulating genetic-information processing. Additionally, bioinformatics analysis suggests that elevated expression of FAM111B augments sensitivity to chemotherapeutic drugs such as cisplatin, paclitaxel, and gemcitabine—indicating potential therapeutic strategies for targeting FAM111B in cancer treatment.

While initially identified as the causative gene of POIKTMP, further research indicated that mutation or overexpression of FAM111B was linked to various types of cancers [3, 4, 6, 12, 17, 18]. We herein observed that FAM111B knockdown attenuated tumor growth both in vitro and in vivo in ovarian cancer, with results aligning with a previous study that illustrated the impact of FAM111B suppression on tumor growth, G1/S phase cell-cycle arrest, and apoptosis in ovarian cancer [18]. In the context of our study, MYC was identified as a key downstream molecular target distinct from AKT as reported in other studies [7, 18]. Further investigation is warranted to explore the interplay between these two pathways. Consequently, elevated expression of FAM111B, acting as an oncogene, is acknowledged to facilitate malignant biological mechanisms in ovarian cancer.

To elucidate the regulatory mechanism of FAM111B, we assessed its influence on MYC expression. The downregulation of FAM111B in ovarian cancer cells resulted in a reduction in MYC expression levels; and subsequent rescue experiments revealed that the overexpression of MYC significantly affected the FAM111B phenotype, leading to the restoration of cellular viability, proliferation, migration, and invasion. Hence, we hypothesized that MYC likely serves as a critical component in the subsequent regulatory cascade of FAM111B. Additionally, our protein transcriptomic investigation showed that the FAM111B-MYC axis facilitated oncogenic proliferation by modulating genetic-information processing. Notably, FAM111B has been implicated in DNA replication and DNA damage-repair pathways in esophageal cancer [19]. The MYC proto-oncogene encodes a DNA-binding factor that governs the transcriptional regulation of genetic information [20], and MYC protein is essential for DNA replication, with its dysregulation through abnormal activation contributing to tumor development [21]. We herein identified a novel role for FAM111B in regulating genetic-information processing by modulating the MYC pathway. Previous investigations have indicated that the phenotypic effects of FAM111B encompass energy metabolism and cellular adaptability, actions congruent with established functions of MYC in energy metabolism, tumor maintenance, proliferative inhibition, senescence, and apoptosis [12, 16, 22]. Therefore, MYC may serve as a crucial signaling mediator in the oncogenic effects that depend upon FAM111B, but this hypothesis necessitates additional investigation.

Other regulatory mechanisms of FAM111B are anticipated to participate in its oncogenic functions across various cancer types. For example, in lung adenocarcinoma, FAM111B modulates the expression of BCL2 and BAG3 to facilitate cell proliferation [11], and FAM111B exhibits peak expression levels during the early G0 phase and late S phase of the cell cycle. The gene also engages in regulating cell-cycle progression through a mechanism that is dependent upon cyclin D1-CDK4 [8, 12]. Moreover, FAM111B is involved in facilitating the development of hepatoma cells via the activation of the p53 pathway [8]. Therefore, it is likely that the FAM111B regulatory network extends to various downstream pathways, potentially augmenting its capacity to facilitate malignancy.

**Fig. 9:**
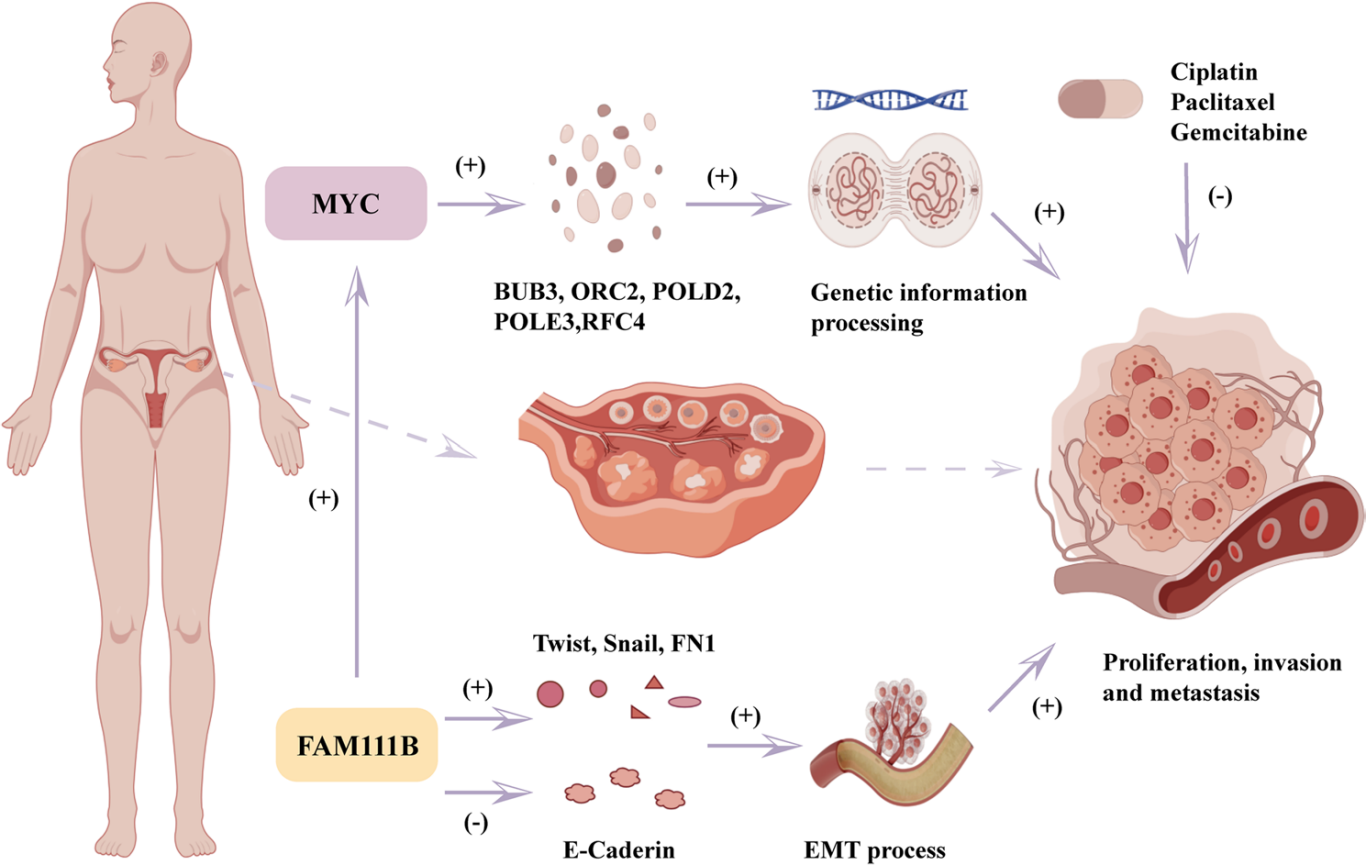
Figdraw diagram depicting the function of FAM111B in ovarian cancer

The findings of this study indicate the potential therapeutic significance of inhibiting FAM111B in cancers that exhibit elevated FAM111B expression (Fig. 9). Although there are currently no documented examples of targeted FAM111B inhibitors, various approaches may be pursued to promote the development of effective treatments. Upstream of FAM111B, a combination of metformin and aspirin has been demonstrated to markedly reduce FAM111B levels in pancreatic cancer cells, with “cholesterol biosynthesis,” “G1/S checkpoint regulation,” and “axon guidance” signaling pathways exhibiting significant enrichment [23]. We therefore know of a range of compounds that show potential efficacy in targeting FAM111B. Furthermore, in WM35 cells, C646, a transcriptional co-activator and p300/CBP inhibitor, reduced the expression of FAM111B in a manner independent of TP53 [24]. Hence, it is plausible that FAM111B may impact the regulation of chromatin assembly and activation of the DNA-repair pathway via the p300/CBP complex, indicating a potential benefit of targeting FAM111B indirectly by using inhibitors of p300/CBP. Additionally, our findings suggest that MYC would be a viable synthetic lethal target of FAM111B, given that MYC (like other transcription factors) is considered a non-drug target at the molecular level [21, 25]. Nevertheless, it has been reported that small molecules such as 10,058-F4 and 10,074-G5 have the ability to selectively inhibit the interaction between MYC and MAX, thereby indirectly suppressing the activity of MYC [26–28]. However, as a result of their low efficacy and rapid degradation rate, these inhibitors have thus far only been utilized in pre-clinical research.

Additionally, our research findings indicated a positive correlation between FAM111B and BRCA1/BRCA2. It is widely recognized in the field of synthetic lethality that molecularly targeted therapies such as PARP inhibitors possess the potential to greatly extend the progression-free and overall survival rates for individuals with BRCA1- or BRCA2-mutant ovarian cancer [29–31]. Hence, we hypothesize that PARP inhibitors or combination therapies should yield significant therapeutic benefits for individuals exhibiting elevated levels of FAM111B expression. Our analysis further suggests that FAM111B exhibits sensitivity to conventional chemotherapeutic agents such as cisplatin, paclitaxel, and gemcitabine. Consequently, further validation of these compounds and molecules is warranted to explore novel targeted therapeutic approaches for ovarian cancer characterized by high FAM111B expression.

## Conclusions

The present data indicate that FAM111B plays a central role in facilitating malignant cellular processes such as proliferation, migration, invasion, and the EMT in vitro, as well as in promoting tumor growth in vivo. Elevated levels of FAM111B were also correlated with a negative prognosis in patients with serous ovarian cancer. This study additionally revealed that silencing FAM111B led to a reduction in MYC expression and that overexpression of MYC reversed the phenotypic effects of FAM111B silencing. The findings from protein transcriptomic and bioinformatics analysis show that FAM111B portrays a regulatory role in the modulation of MYC activity and thereby influences genetic-information processing. We propose that targeting FAM111B gene holds potential therapeutic implications for ovarian cancer.

## Supplementary Information

Supplementary Material 1.

Supplementary Material 2.

Supplementary Material 3.

## Data Availability

All data generated or analyzed during this study has been included in this article.
