# Supplementary figures and images for: FAM111B knockdown attenuates tumorigenesis of ovarian cancer via the downregulation of MYC
Source: BMC Cancer. 2025 Aug 9;25:1290. doi: 10.1186/s12885-025-14740-6 (PMC12335066; doi:10.1186/s12885-025-14740-6)

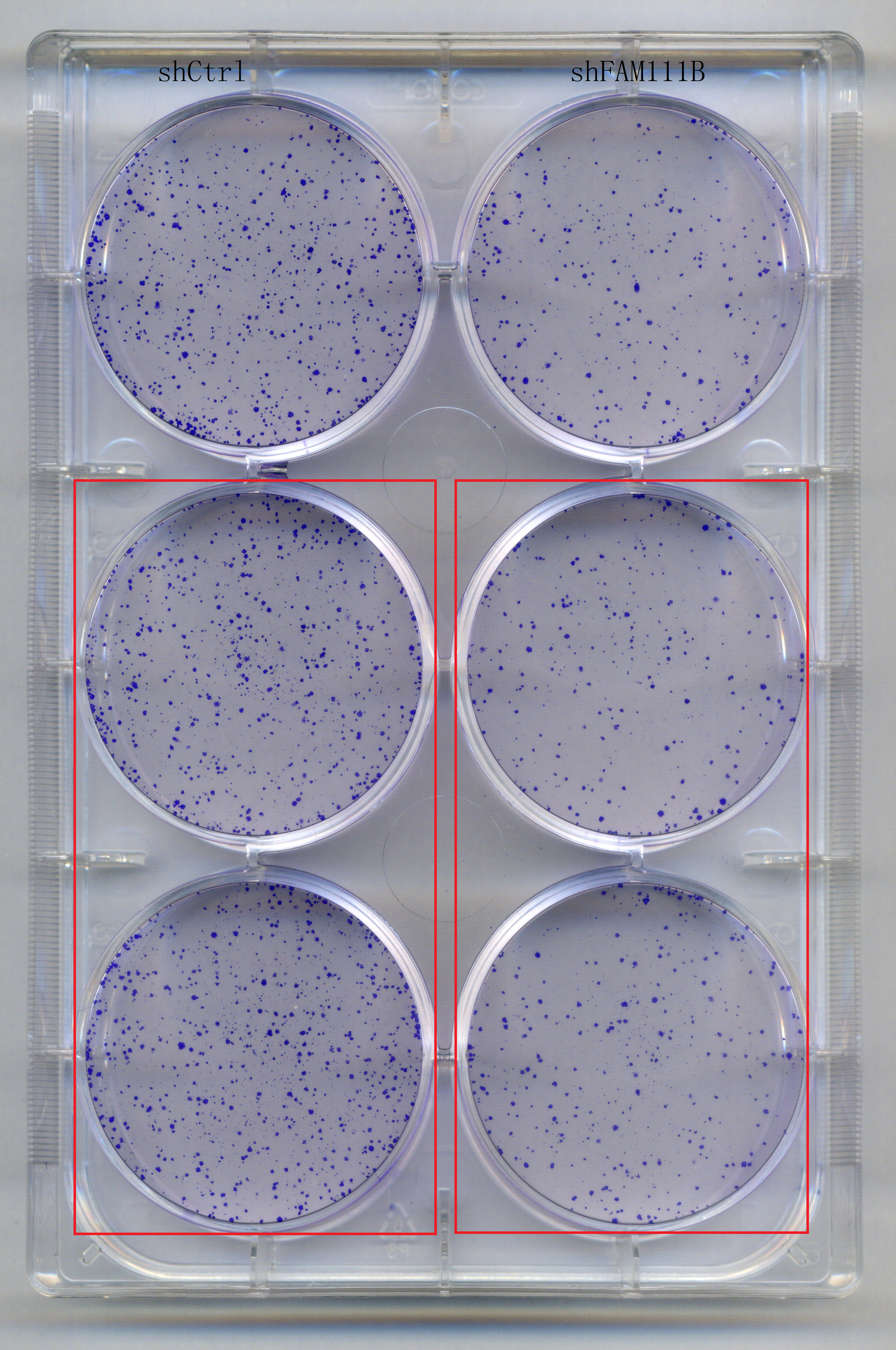

Supplement: Supplementary file 2 — Supplementary Material 2. [file 12885_2025_14740_MOESM2_ESM.zip › Raw data/Raw data of colony formation assay/A2780 shCtrl and shFAM111B.tif]

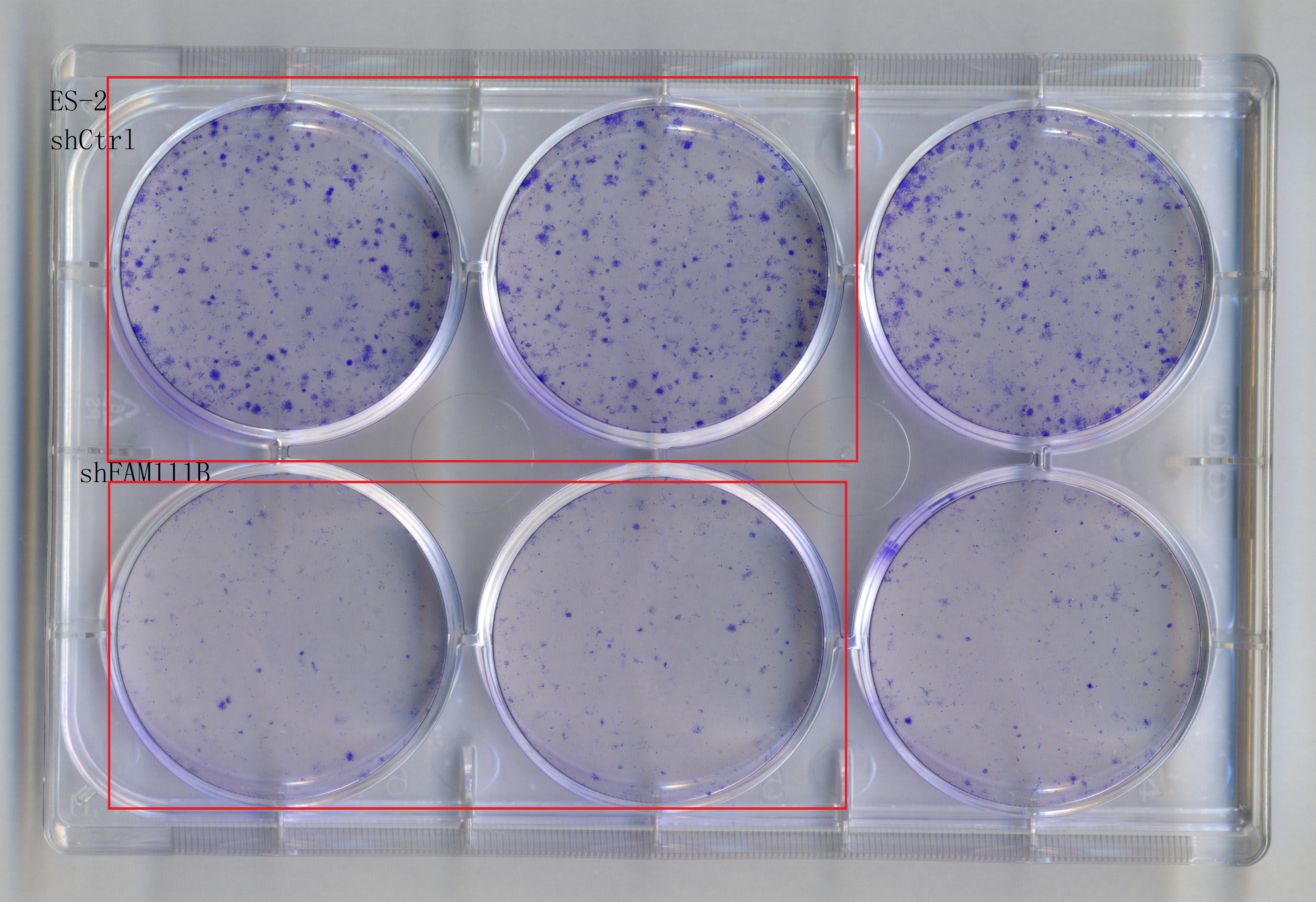

Supplement: Supplementary file 2 — Supplementary Material 2. [file 12885_2025_14740_MOESM2_ESM.zip › Raw data/Raw data of colony formation assay/ES-2 shCtrl and shFAM111B.tif]

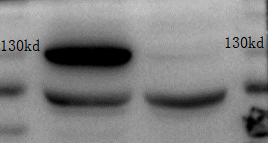

Supplement: Supplementary file 2 — Supplementary Material 2. [file 12885_2025_14740_MOESM2_ESM.zip › Raw data/Raw data of WB/Raw data of WB Fig 1A and 1B/Fig.1A FAM111B of ES-2.tif]

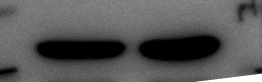

Supplement: Supplementary file 2 — Supplementary Material 2. [file 12885_2025_14740_MOESM2_ESM.zip › Raw data/Raw data of WB/Raw data of WB Fig 1A and 1B/Fig.1A GAPDH of ES-2.Tif]

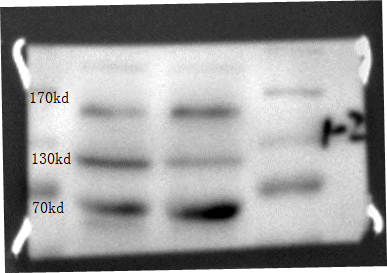

Supplement: Supplementary file 2 — Supplementary Material 2. [file 12885_2025_14740_MOESM2_ESM.zip › Raw data/Raw data of WB/Raw data of WB Fig 1A and 1B/Fig.1B FAM111B of A2780.tif]

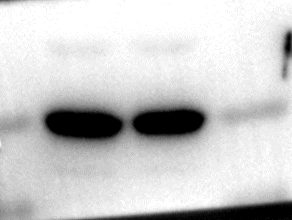

Supplement: Supplementary file 2 — Supplementary Material 2. [file 12885_2025_14740_MOESM2_ESM.zip › Raw data/Raw data of WB/Raw data of WB Fig 1A and 1B/Fig.1B GAPDH of A2780.Tif]

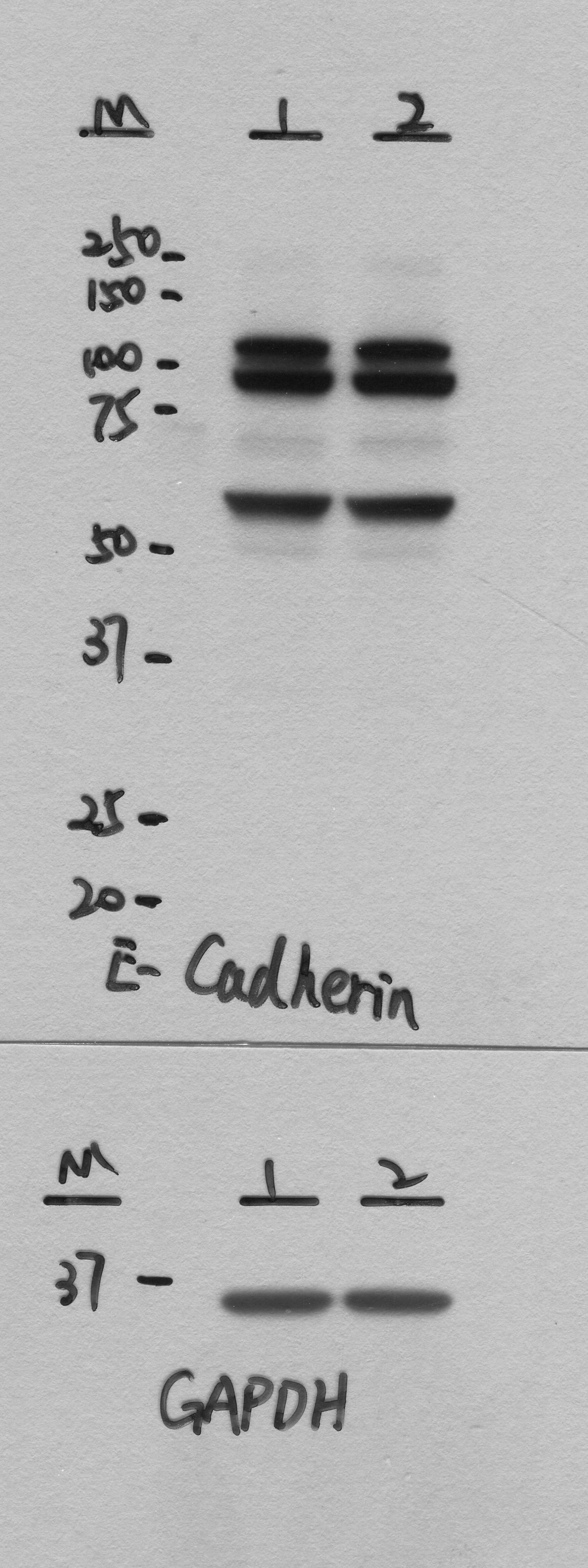

Supplement: Supplementary file 2 — Supplementary Material 2. [file 12885_2025_14740_MOESM2_ESM.zip › Raw data/Raw data of WB/Raw data of WB Fig 2E/A2780/Fig.2E E-Cadherin of A2780.jpg]

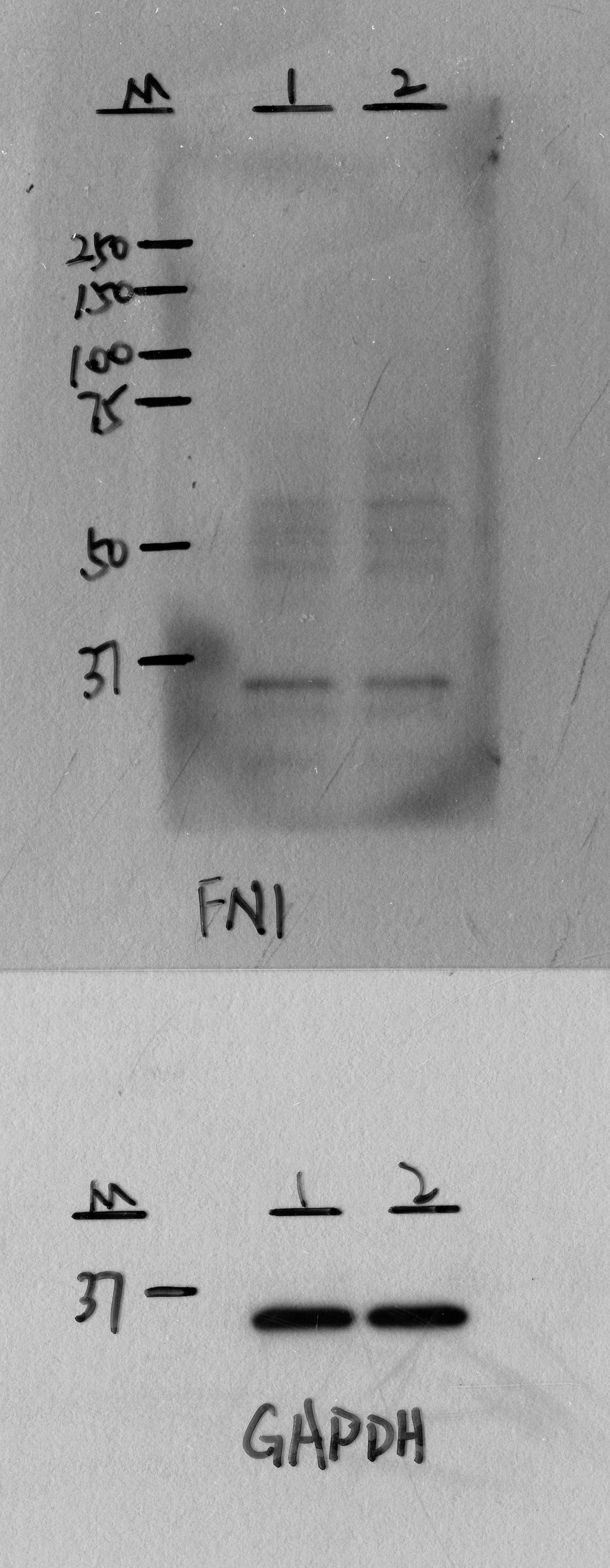

Supplement: Supplementary file 2 — Supplementary Material 2. [file 12885_2025_14740_MOESM2_ESM.zip › Raw data/Raw data of WB/Raw data of WB Fig 2E/A2780/Fig.2E FN1 of A2780.jpg]

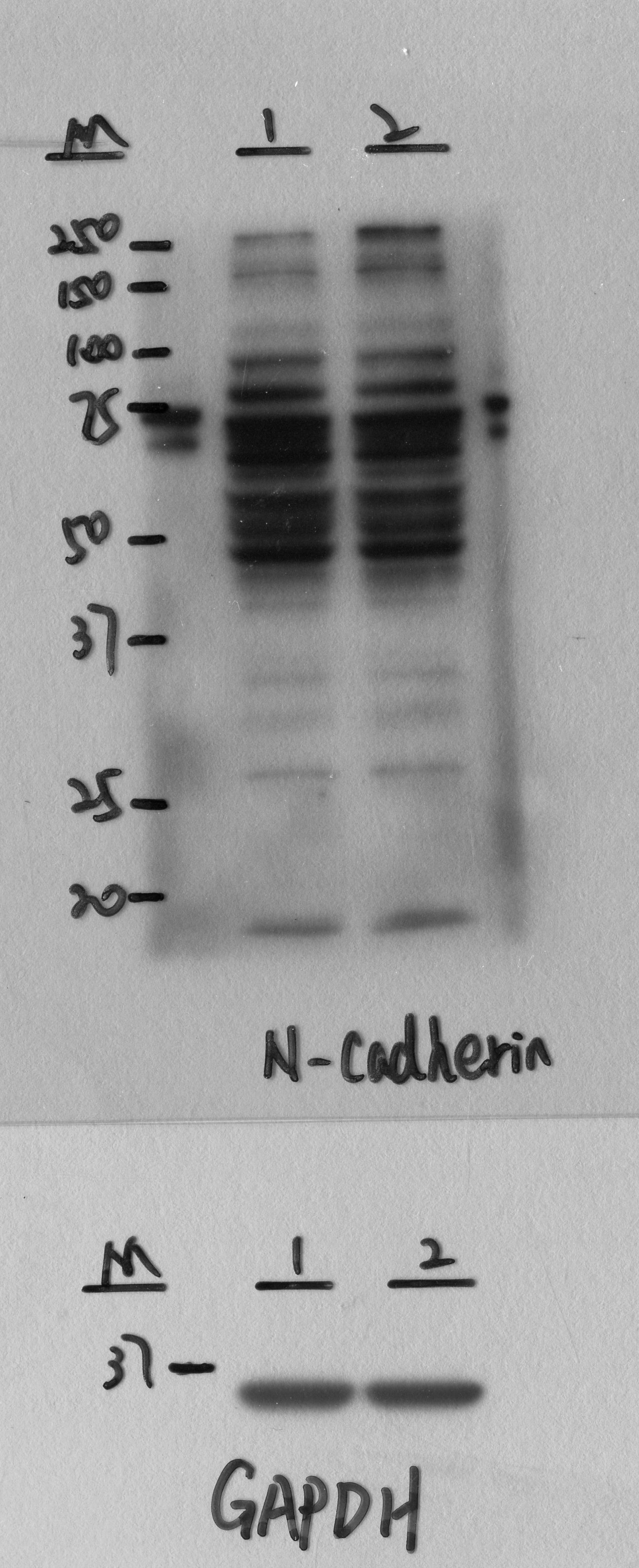

Supplement: Supplementary file 2 — Supplementary Material 2. [file 12885_2025_14740_MOESM2_ESM.zip › Raw data/Raw data of WB/Raw data of WB Fig 2E/A2780/Fig.2E N-Cadherin of A2780.jpg]

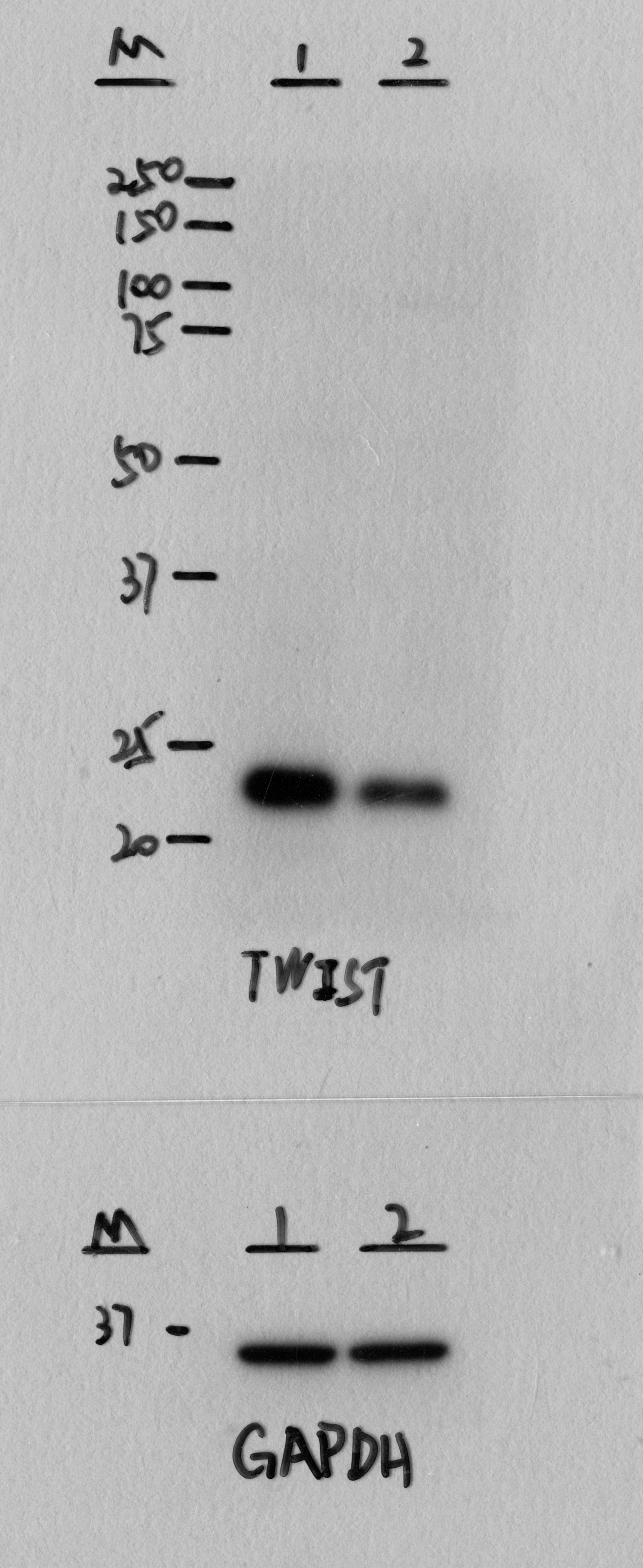

Supplement: Supplementary file 2 — Supplementary Material 2. [file 12885_2025_14740_MOESM2_ESM.zip › Raw data/Raw data of WB/Raw data of WB Fig 2E/A2780/Fig.2E TWIST of A2780.jpg]

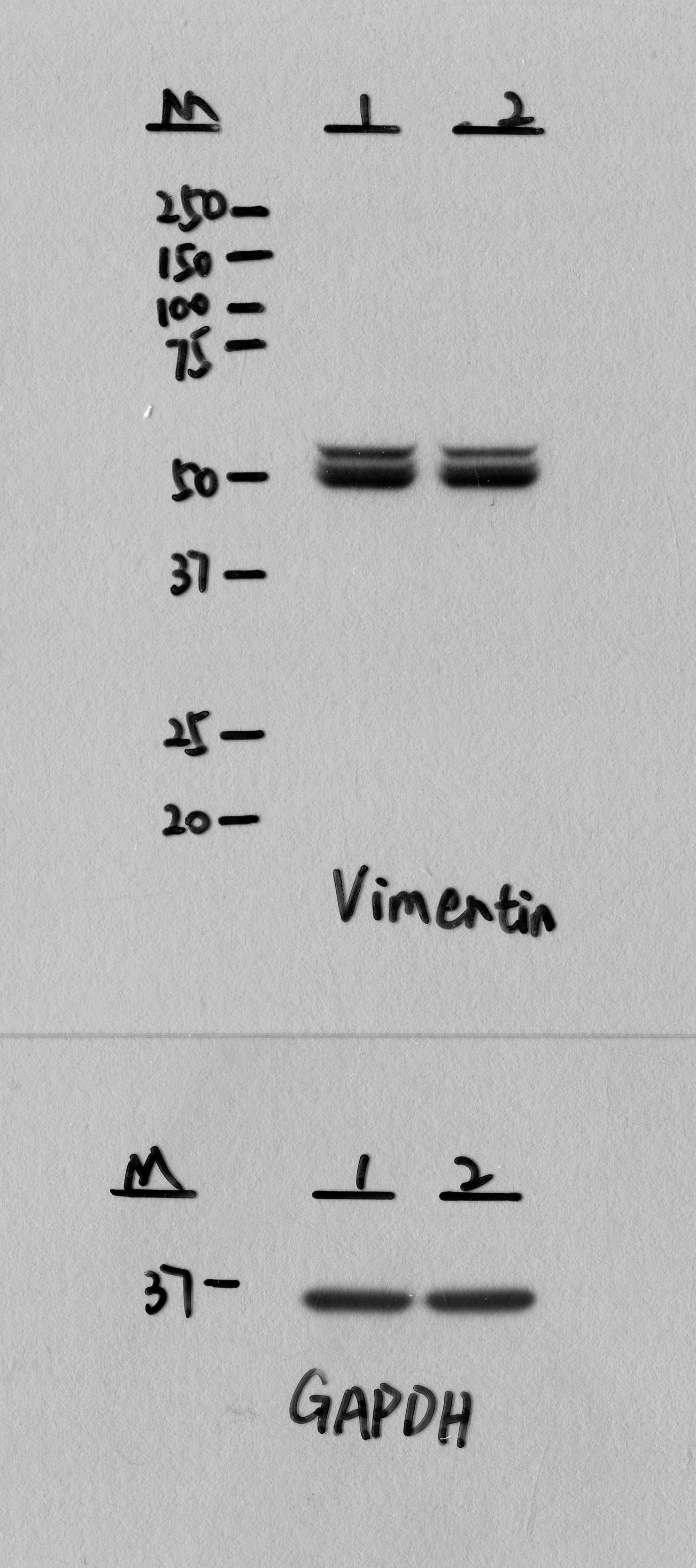

Supplement: Supplementary file 2 — Supplementary Material 2. [file 12885_2025_14740_MOESM2_ESM.zip › Raw data/Raw data of WB/Raw data of WB Fig 2E/A2780/Fig.2E Vimentin of A2780.jpg]

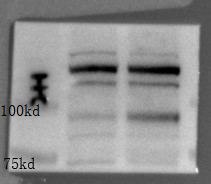

Supplement: Supplementary file 2 — Supplementary Material 2. [file 12885_2025_14740_MOESM2_ESM.zip › Raw data/Raw data of WB/Raw data of WB Fig 2E/ES2/Fig.2E E-Cadherin of ES2.tif]

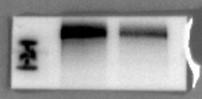

Supplement: Supplementary file 2 — Supplementary Material 2. [file 12885_2025_14740_MOESM2_ESM.zip › Raw data/Raw data of WB/Raw data of WB Fig 2E/ES2/Fig.2E FN1 of ES2.jpg]

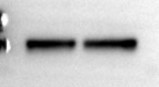

Supplement: Supplementary file 2 — Supplementary Material 2. [file 12885_2025_14740_MOESM2_ESM.zip › Raw data/Raw data of WB/Raw data of WB Fig 2E/ES2/Fig.2E GAPDH of ES-2.jpg]

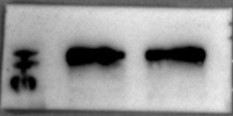

Supplement: Supplementary file 2 — Supplementary Material 2. [file 12885_2025_14740_MOESM2_ESM.zip › Raw data/Raw data of WB/Raw data of WB Fig 2E/ES2/Fig.2E N-Cadherin of ES-2.jpg]

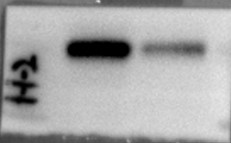

Supplement: Supplementary file 2 — Supplementary Material 2. [file 12885_2025_14740_MOESM2_ESM.zip › Raw data/Raw data of WB/Raw data of WB Fig 2E/ES2/Fig.2E Twist of ES2.jpg]

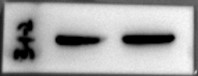

Supplement: Supplementary file 2 — Supplementary Material 2. [file 12885_2025_14740_MOESM2_ESM.zip › Raw data/Raw data of WB/Raw data of WB Fig 2E/ES2/Fig.2E Vimentin of ES2.jpg]

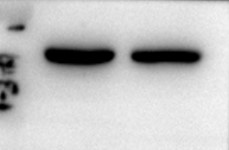

Supplement: Supplementary file 2 — Supplementary Material 2. [file 12885_2025_14740_MOESM2_ESM.zip › Raw data/Raw data of WB/Raw data of WB Fig 3A and 3B/Fig.3A GAPDH1 of ES2.jpg]

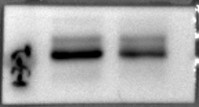

Supplement: Supplementary file 2 — Supplementary Material 2. [file 12885_2025_14740_MOESM2_ESM.zip › Raw data/Raw data of WB/Raw data of WB Fig 3A and 3B/Fig.3A MYC low-expression of ES2.jpg]

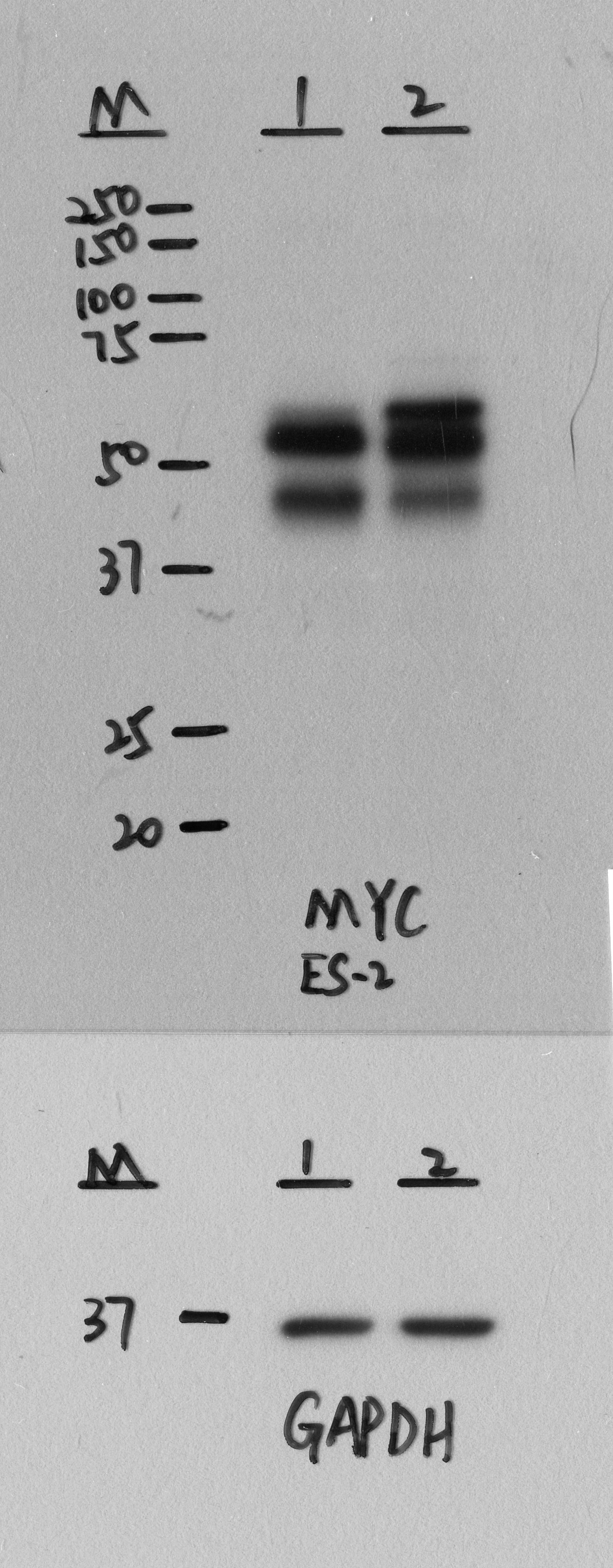

Supplement: Supplementary file 2 — Supplementary Material 2. [file 12885_2025_14740_MOESM2_ESM.zip › Raw data/Raw data of WB/Raw data of WB Fig 3A and 3B/Fig.3A MYC overexpression of ES2.jpg]

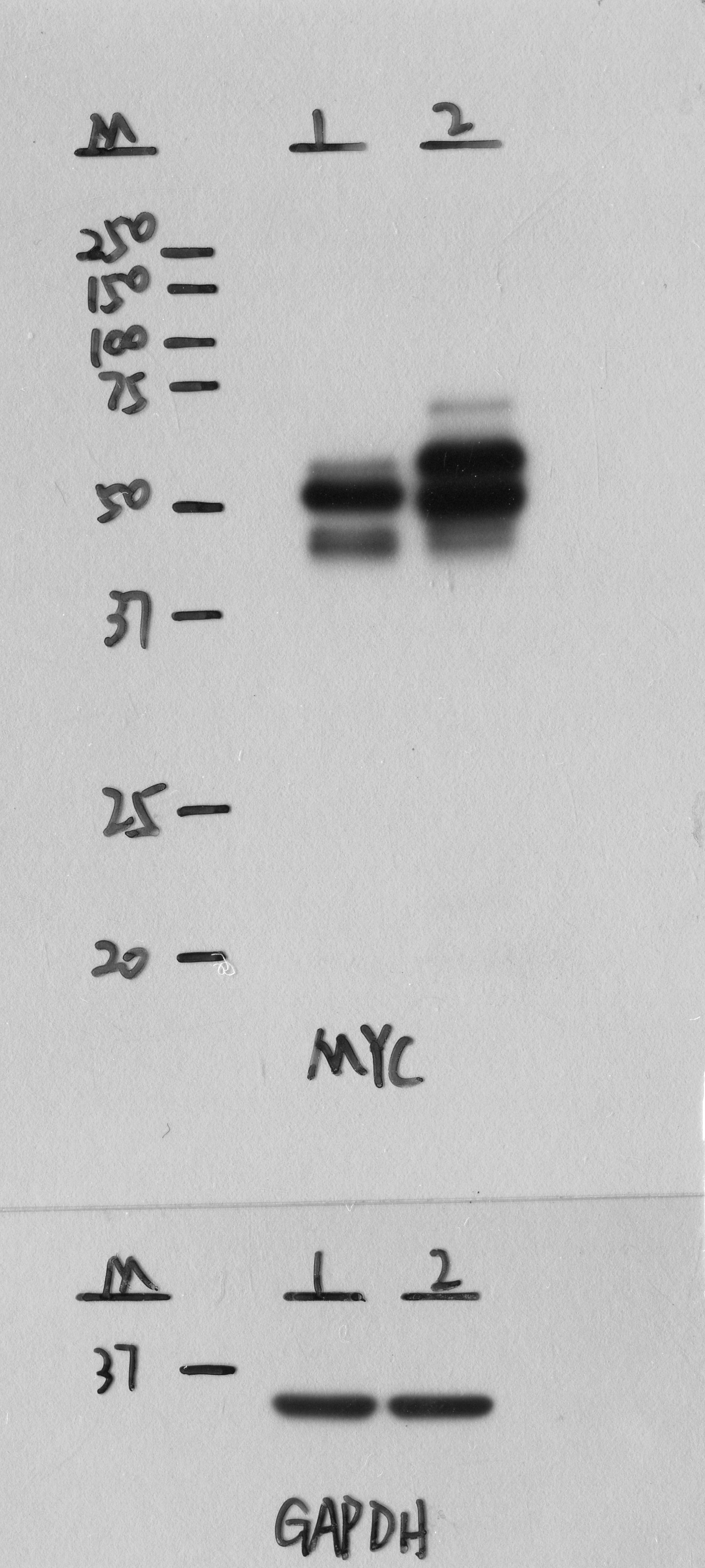

Supplement: Supplementary file 2 — Supplementary Material 2. [file 12885_2025_14740_MOESM2_ESM.zip › Raw data/Raw data of WB/Raw data of WB Fig 3A and 3B/Fig.3B MYC overexpression of A2780.jpg]

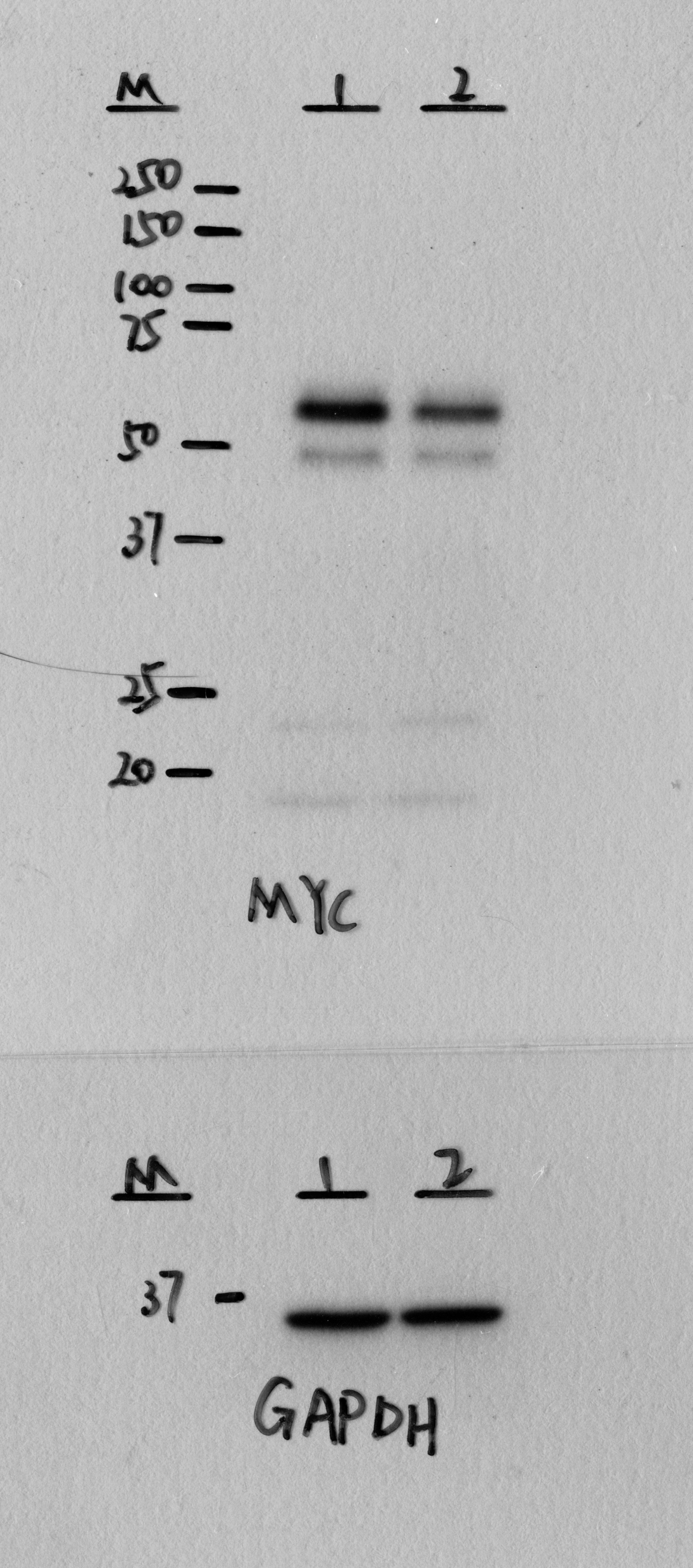

Supplement: Supplementary file 2 — Supplementary Material 2. [file 12885_2025_14740_MOESM2_ESM.zip › Raw data/Raw data of WB/Raw data of WB Fig 3A and 3B/Fig.3B MYClow-expression of A2780.jpg]
