# Supplementary material for: FAM111B knockdown attenuates tumorigenesis of ovarian cancer via the downregulation of MYC
Source: BMC Cancer. 2025 Aug 9;25:1290. doi: 10.1186/s12885-025-14740-6 (PMC12335066; doi:10.1186/s12885-025-14740-6)
Supplement: Supplementary file 3 — Supplementary Material 3. [file 12885_2025_14740_MOESM3_ESM.pdf]

CERTIFICATE

OF ENGLISH LANGUAGE EDITING

FAM111B knockdown attenuates tumorigenesis of ovarian cancer via the downregulation of MYC

Background Ovarian cancer, a prevalent malignancy with the highest fatality rate among gynecological cancers, continues to face challenges in the development of effectively targeted therapeutic approaches. While the FAM111B gene has been implicated in various cancer types, its specific role in ovarian cancer remains poorly understood.
Methods The ES2 and A2780 ovarian cell lines were exploited to explore the cellular proliferation, migration, invasion, and epithelial-mesenchymal transition (EMT) in FAM111B knockdown experiments. We constructed a mouse tumor model to investigate the impact of FAM111B silencing in vivo; employed tissue microarray to explore the prognostic value of different FAM111B expression levels; and applied western-blot assay, MYC-overexpression rescue experiments, protein transcriptomics, and bioinformatic analysis to examine the ...

This document certifies that the manuscript listed above was copy edited for English language by LetPub, with regard to grammar, punctuation, spelling, and clarity. Documents receiving this certification should be regarded as having undergone professional editorial revision for English language before submission. However, the authors may accept or reject LetPub's suggestions and changes at their own discretion and LetPub does not have editorial control over the submitted documents. Submitted documents may have new text that was not provided to LetPub for review. Please use the verification link below to determine the validity of the submitted version.

September 18, 2024

Date of Revision

LetPub

LetPub is an author service brand owned and operated by Accdon LLC.
Tel: 1-781-202-9968 Email: info@accdon.com
Address: 400 Fifth Ave, Suite 530, Waltham, MA 02451, United States

This manuscript has been individually edited for grammar, punctuation, spelling, and clarity. You may verify the authenticity of this certificate on our website (https://www.letpub.com/editorial-certificate) at any time using this manuscript's unique code: PR_240912Z148F.

© 2024 Accdon, LLC.
